# Squamous Cell Carcinoma of the Submandibular Gland With Cutaneous Fistula: A Case Report and Literature Review

**DOI:** 10.7759/cureus.27785

**Published:** 2022-08-08

**Authors:** Ilias Tahiri, Othman El Houari, Taali Loubna, Amal Hajjij, Mohammed Zalagh

**Affiliations:** 1 Otorhinolaryngology, Mohammed VI University of Health Sciences (UM6SS), Casablanca, MAR; 2 Otolaryngology - Head and Neck Surgery, Mohammed VI University of Health Sciences (UM6SS), Casablanca, MAR; 3 Otorhinolaryngology, International Cheikh Khalifa University Hospital, Mohammed Vi University of Health Sciences (UM6SS), Casablanca, MAR; 4 Otolaryngology - Head and Neck Surgery, International Cheikh Khalifa University Hospital, Mohammed Vi University of Health Sciences (UM6SS), Casablanca, MAR

**Keywords:** cancer immunotherapy, squamous cell carcinoma, radiotherapy, rhomboid flap, pet ct scan

## Abstract

Squamous cell carcinoma (SCC) of salivary glands, also referred to as epidermoid carcinoma, is a very rare neoplastic tumor. It occurs as metastasis of a cutaneous or mucosal squamous carcinoma of the head and neck or as a primary SCC. In the latter case, the most known risk factor is previous irradiation to the gland. Common clinical symptoms are represented by cervical swelling and hyposialia. The treatment is essentially surgical, most often supplemented by a radical neck dissection and postoperative radiation therapy.

A 75-year-old male patient with a history of chronic smoking was consulted for a tumefaction in the right submandibular region evolving for three months. No cervical lymphadenopathy in the submandibular and superior jugulo-carotid areas was palpable. CT scan showed an enhancing heterogeneous process of the right cervical region, invading the mylohyoid and stylohyoid muscles. A biopsy-excision of the lesion has shown a keratinizing tumor with cytonuclear atypia, consistent with SCC. Radical resection of the mass was associated with the removal of the infiltrated skin. The cutaneous defect was repaired with a rhomboid flap. The patient was started on sessions of adjuvant radiotherapy and chemotherapy. Eight months postoperatively, the patient came for follow-up, with no signs of local disease.

The EGFR protein is found in ~70% of salivary neoplasms and is considered as a factor of poor prognosis and rapid proliferation. PET CT is currently the best examination to detect the existence of a concomitant malignant lesion. Diagnosis of primary SCC (PSCC) of the submandibular gland is made on histopathology. Differential diagnoses include mucoepidermoid carcinoma, lymphoepithelial carcinoma and submandibular metaplasia. There is an increased prevalence of nodal involvement in the PSCC, which justifies neck dissection (regions I, II and III of the neck). The RAS mutation leading to resistance to anti-EGFR therapies may be assessed. This would allow for a treatment depending on molecular features for metastatic PSCCs.

PSCC of major salivary glands is a very rare lesion with local and general aggressiveness. The diagnosis is based on a combination of clinical examination, MRI, fine needle aspiration and histological examination. Immunotherapy constitutes a ground of research to treat metastatic and advanced pathological cases.

## Introduction

Most neoplastic lesions of the major salivary gland are benign. The malignant lesions are rare and include mainly mucoepidermoid carcinoma [1]. Squamous cell carcinoma (SCC) of salivary glands, also referred to as epidermoid carcinoma, is a very rare malignant tumor. It represents around 1.8 % of all salivary neoplasms and is a proliferation of squamous epidermoid cells [2].

SCC usually develops in mucosal surfaces of the upper aerodigestive tract. It is associated with chronic smoking and alcohol intake. The SCC of the major salivary glands, do not present the same pathophysiological mechanisms. It mostly occurs as metastasis of a cutaneous or mucosal squamous carcinoma of the head and neck. The most known risk factor is previous irradiation to the gland [3]. Common clinical symptoms are cervical swelling and hyposialia. At the time of diagnosis, cervical node metastases have an incidence of 21% to 45% [4].

Histologically, the presence of squamous cells that are round to polygonal with eosinophilic cytoplasm, is the hallmark feature of this lesion. Immunohistochemistry (IHC) is useful for determining positive CK and p63 markers [5]. The treatment is determined after a multidisciplinary consultation meeting. It is essentially surgical, most often supplemented by a radical neck dissection and postoperative radiation therapy. Surgical removal is often disfiguring, given the difficulty to achieve resection in free margins. We report the case of a rare case of SCC of the submandibular gland with a sialo-cutaneous fistula and a review of the previously reported cases of SCCs of major salivary glands in the literature.

## Case presentation

We report a case of a 75-year-old male patient with a history of chronic, with no hypertension, diabetes or general illness. He consulted on February 2021 in the Otorhinolaryngology Department at the Cheikh Khalifa International University Hospital (CKIUH). He presented a swelling in the right submandibular region evolving for three months. The patient did not report any symptoms of dyspnea, dysphonia, or epistaxis. The submandibular mass was hard, firm and was accompanied by local inflammation.

There was a fistulous orifice adjacent to the mass, discharging a mucoid fluid at palpation. Intraoral examination showed normal-aspect mucosa. The floor of the mouth, palatine tonsils and oropharynx were not the sites of any suspicious lesion. No cervical lymphadenopathy in the submandibular and superior jugulo-carotid areas was palpable. The patient underwent a nasofibroscopy to explore the rest of the upper aerodigestive tract. No synchronous malignancy was ruled out through the examination.

**Figure 1:**
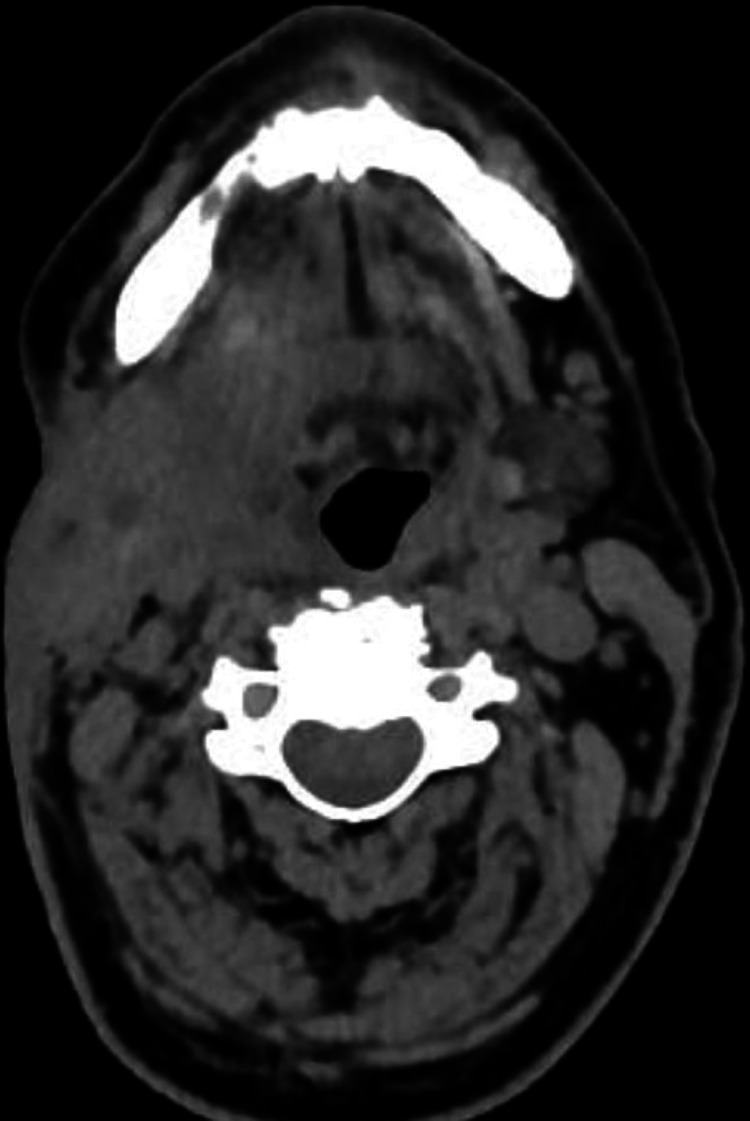
Scannographic presentation of the right submandibular swelling (heterogeneous mass with irregular contours extending into the right parapharyngeal space and palatine fossa).

**Figure 2:**
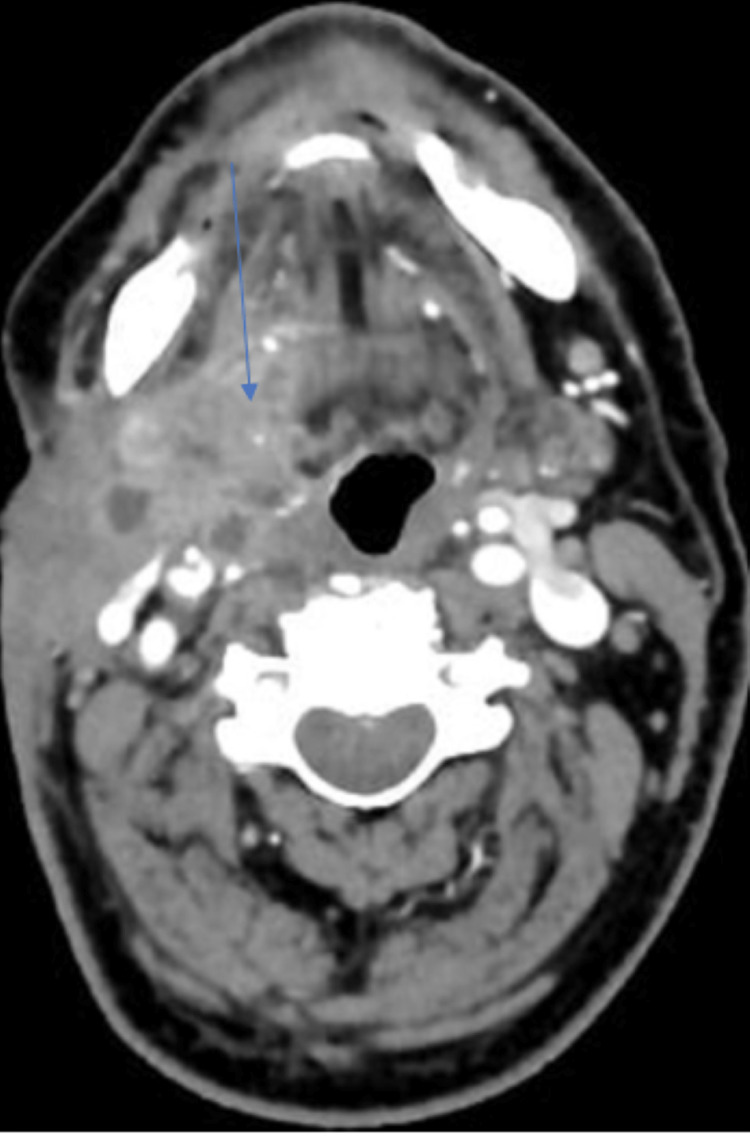
Scannographic presentation of the right submandibular mass showing high enhancement after injection of the contrast product. Contrasted areas (blue arrow)

The cervical ultrasonography revealed a hypoechoic lesion of the right submandibular gland, highly suggestive of neoplasia. CT scan showed an enhancing heterogeneous process of the right cervical region of 4×3.2×4.2 cm, causing a defect of the skin, invading the mylohyoid and stylohyoid muscles, coming into close contact with the right parotid gland and the jugular vein (Figures 1, 2). There were several suspicious lymph nodes of the submental (I), superior and middle jugulo-digastric areas (II and III) of the right neck.

**Figure 3:**
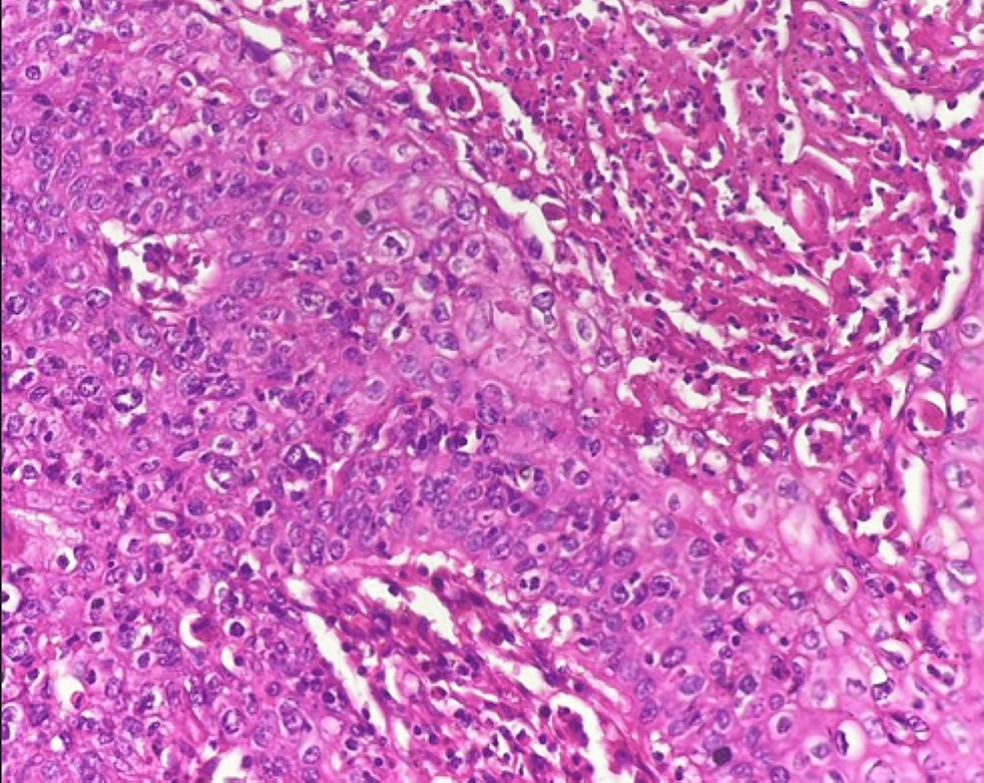
Tumoral proliferation made of squamous cells with cytonuclear atypias and numerous mitoses, consistent with SCC. Hematoxylin-eosin stain, original magnification *40.

**Figure 4:**
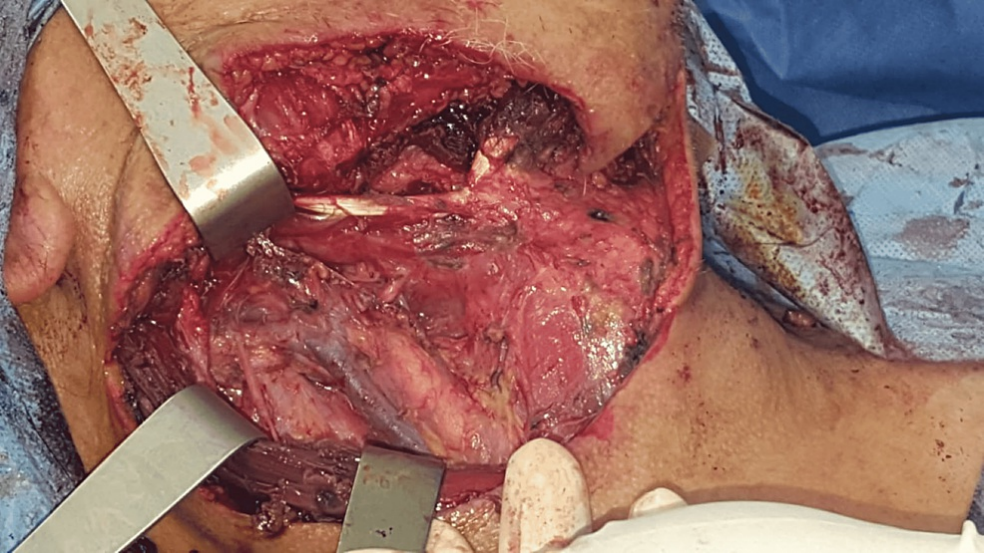
Post-operative aspect after complete resection of the lesion.

A biopsy excision of the lesion has shown a keratinizing tumor with cytonuclear atypias and numerous mitoses. The chorion was infiltrated by spans, cords and nests of the carcinomatous lesion (Figure 3). Diagnosis of a moderately differentiated SCC of the submandibular gland was made. The objective of the therapeutic protocol was to avoid revision surgery and allow for complete removal of the lesion. After a multidisciplinary consultation meeting, we decided to perform a neck dissection comprising levels I, II and III (Figure 4). The neck dissection material showed no signs of malignancy.

**Figure 5:**
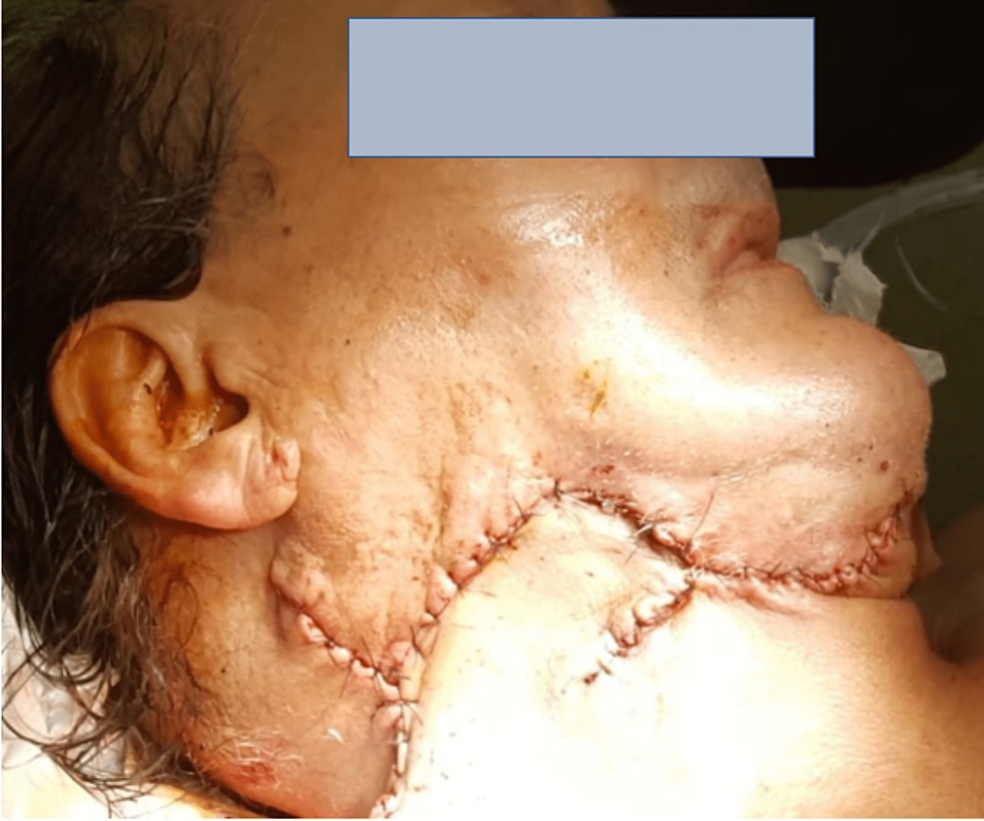
Rhomboid flap allowing skin closure.

Radical resection of the mass was associated with the removal of the infiltrated opposite skin. The cutaneous defect was repaired with a rhomboid flap (Figure 5).

The final pathological examination confirmed the diagnosis of SCC of the submandibular gland. As the deep margins of the tumor were invaded, our patient underwent revision surgery. Multiple biopsies of the suspected site confirmed the complete removal of the carcinoma after a histopathological study. The patient was started on 35 sessions of adjuvant radiotherapy (70 Gy). The treatment was completed by 10 courses of cisplatin-based chemotherapy. Eight months postoperatively, the patient came for follow-up, with no signs of cervical swelling, pain or hyposialia. Nasofibroscopy and control CT scan showed no sign of local disease.

## Discussion

Primary SCC (PSCC) of the submandibular gland is a particular entity of the SCC of major salivary glands. The latter represents ~2 % of all malignant salivary gland tumors and arises most of the time in the parotid (~80%). Patients with PSCC present a poor overall survival outcome, approaching ~50% at 5 years [6]. This can be related to the highly recurrent nature of the disease (~66% in the first year) [7]. Studies have shown that patients with primary and metastatic SCC of the major salivary glands have both the same prognosis [6]. Also, the presence of lung metastases is a life-threatening prognostic factor [7].

The EGFR protein is found in ~70% of salivary neoplasms and is considered a factor of poor prognosis and rapid proliferation. It has been demonstrated that the Wnt/β-catenin pathway induces tumor development by increasing self-renewal. Indeed, Wnt glycoprotein activation permits signal transduction. This generates an elevation of the intracellular level of the β-catenin complex. The nuclear translocation of β-catenin is then followed by the transcription of multiple targeted genes (i.e., Cyclin D1). Loss of membrane β-catenin has been shown to distort cell adhesion, allowing for migration and metastatic invasion. This is combined with the aggressive nature of the tumor and its drug resistance [8].

PSCC of the submandibular gland remains a diagnosis of exclusion. Differential diagnoses have to be excluded: (1) metastasis from a distant primary site, (2) mucoepidermoid carcinoma, and (3) cutaneous SCC extending locally. It is difficult in histopathology to differentiate between a PSCC and a secondary or metastatic localization. Indeed, both are keratinizing lesions with no specific features for the primary tumor. The radiological investigations are necessary due to the fact that the identification of the site of origin is often not obvious at the time of presentation. PET CT is currently the best examination to detect the existence of a concomitant malignant lesion [9]. In the case of our patient, PET CT evaluations have not revealed any secondary metastatic site.

This disease occurs mainly in men with a male to female ratio of 2:1. The highest incidence was found to be in the sixth and seventh decades of life [9]. This correlates to our case study. The main described risk factor is cervical radiotherapy, with an onset interval of 10 years [9]. In our case, there was no history of irradiation to salivary glands. Snuff, tobacco, and lime chewing are incriminated in the SCC of the oral cavity but there is no proof for the major salivary gland SCC [10]. The early diagnosis appears to be an important prognostic factor [11].

An accurate initial imaging assessment is essential for staging the tumor and deciding the therapeutic approach. Fine-needle aspiration (FNA) biopsy is highly recommended before surgery in order to obtain a reliable histological diagnosis [11]. Diagnosis of PSCC of the submandibular gland is a challenge for histopathologists. Indeed, ductal squamous metaplasia can be a diagnostic trap, especially in patients previously treated for cancer of the head and neck region. In patients treated with radiation therapy, an FNA biopsy of the tumefaction usually reveals squamous cells in the liquid which can confuse the pathologist, leading to a false diagnosis.

For this reason, the biopsy with pathological examination is essential to confirm an SCC lesion [12]. The existence of cytonuclear atypia organized in islands and cords is absent in patients with metaplasia. Also, round and uniform metaplastic ducts, acinar atrophy and infiltrative inflammation are highly suggestive of the PSCC [12]. In the absence of mucin-producing cells and keratinization, negativity for periodic acid Schiff (PAS) helps differentiate between a primary SCC and mucoepidermoid carcinoma. Also, the presence of another SCC of the upper aerodigestive tract or skin in the history of the patient excludes the diagnosis of a primary tumor. Microscopic features such as oval-shaped cells, a large number of tonofilaments, and desmosome-like cytoplasmic structures, when positive, are consistent with the diagnosis of SCC. Another differential diagnosis that we had to rule out was lymphoepithelial carcinoma. IHC for Epstein-Barr virus was negative, allowing for the exclusion of the latter diagnosis [13].

Studies have shown a very high incidence of local recurrences with exclusive surgery in SCC of the parotid (~50%) and submandibular gland (~60%) [13]. The treatment objective is to avoid such post-operative issues. Therefore, it is multimodal and includes multiple specialties to provide the best management plan. It relies on radical surgery and adjuvant therapy (radiotherapy and chemotherapy). In our case, there was no evidence of local recurrence at the eight months follow-up. For patients with parotid gland SCC, a total parotidectomy with preservation of the facial nerve should be performed. Indeed, in tumors without perineural invasion, nerve-sparing procedures have demonstrated disease control two years post-operatively [14].

There is an increased prevalence of nodal involvement in the PSCC, which justifies neck dissection (regions I, II and III) [14]. As infra-clinical lymphadenopathies cannot be seen by MRI, an ipsilateral modified radical neck dissection is used to detect occult lymph node metastases. Bilateral lymph nodes dissection should be indicated only for cases of clinically palpable bilateral lymphadenopathies of the neck. Postoperative management may combine radiotherapy with cisplatin chemotherapy (CTH). The usual dose is 100 mg/mq with a one-month interval between cures. Whether to consider exclusive radiotherapy or chemotherapy depends on the general condition of the patient, his age and possible associated pathologies (heart disease, hypertension, diabetes...) [15]. Early hematogenous dissemination is a significant challenge for treating SCCs of the major salivary glands. Local recurrence can be associated with distant metastasis or occur alone. This underlines the major role of local control in disease treatment.

Metastatic patients presenting PSCC of the major salivary glands have a poor overall-survival outcome. Indeed, such disseminated tumors are characterized by chemo-resistance and fast growth of carcinomatous lesions. Nevertheless, there is a lack of data and guidelines allowing for treating these cases in a systematic way [15]. Cisplatin, 5-fluoro-uracile, and anthracyclines are the main molecules used in salivary gland tumors including PSCCs. In our case, there was no metastasis at the time of diagnosis.

Given the role of the EGFR protein and the Wnt/β-catenin pathway in the carcinogenesis of the SCC of major salivary glands [16], immunotherapy may be a promising ground for research as it comes to metastatic disease. The functioning of the immune system towards carcinomas of the salivary glands is still not completely elucidated. Prospective studies may evaluate the involvement of EGFR and Wnt proteins in tumor pathogenesis. The RAS mutation leading to resistance to anti-EGFR therapies may be assessed. This would allow for a treatment depending on molecular features for metastatic PSCCs [16].

## Conclusions

PSCC of major salivary glands is a very rare lesion with local and general aggressiveness. The diagnosis of this pathology is based on a combination of clinical examination, MRI, fine needle aspiration and histological examination.

PET CT scan allows evaluation of tumor extension to guide therapeutic management. Surgery is the cornerstone of treatment, most often supplemented by radiotherapy and chemotherapy. Immunotherapy constitutes a ground of research to treat metastatic and advanced pathological cases.
